# Concomitant use of interleukin-2 and tacrolimus suppresses follicular helper T cell proportion and exerts therapeutic effect against lupus nephritis in systemic lupus erythematosus-like chronic graft versus host disease

**DOI:** 10.3389/fimmu.2024.1326066

**Published:** 2024-04-11

**Authors:** Yutaro Nasa, Atsushi Satake, Ryohei Tsuji, Ryo Saito, Yukie Tsubokura, Hideaki Yoshimura, Tomoki Ito

**Affiliations:** First Department of Internal Medicine, Kansai Medical University, Osaka, Japan

**Keywords:** interleukin-2, calcineurin inhibitor, regulatory T cell, follicular helper T cell, follicular regulatory T cell, lupus nephritis, chronic graft versus host disease

## Abstract

**Introduction:**

Defective interleukin-2 (IL-2) production contributes to immune system imbalance in patients with systemic erythematosus lupus (SLE). Recent clinical studies suggested that low-dose IL-2 treatment is beneficial for SLE and the therapeutic effect is associated with regulatory T cell (Treg) expansion. Pharmacological calcineurin inhibition induces a reduction in the number of Tregs because they require stimulation of T cell receptor signaling and IL-2 for optimal proliferation. However, the activation of T cell receptor signaling is partially dispensable for the expansion of Tregs, but not for that of conventional T cells if IL-2 is present.

**Aim:**

We examined whether addition of IL-2 restores the Treg proportion even with concurrent use of a calcineurin inhibitor and if the follicular helper T cell (Tfh) proportion is reduced in an SLE-like murine chronic graft versus host disease model.

**Methods:**

Using a parent-into-F1 model, we investigated the effect of IL-2 plus tacrolimus on Treg and Tfh proportions and the therapeutic effect.

**Results:**

Treatment with a combination of IL-2 and tacrolimus significantly delayed the initiation of proteinuria and decreased the urinary protein concentration, whereas tacrolimus or IL-2 monotherapy did not significantly attenuate proteinuria. Phosphorylation of signal transducer and activator of transcription 3, a positive regulator of Tfh differentiation, was reduced by combination treatment, whereas phosphorylation of signal transducer and activator of transcription 5, a negative regulator, was not reduced.

**Conclusion:**

Addition of calcineurin inhibitors as adjunct agents may be beneficial for IL-2-based treatment of lupus nephritis.

## 1 Introduction

Systemic erythematosus lupus (SLE) is characterized by excessive production of nuclear debris because of aberrant and massive apoptotic events. These debris are recognized as foreign by the immune system, leading to abnormal antigen presentation that induces the loss of B and T cell tolerance (1, 2). This loss of tolerance leads to T cell hyperactivation, triggering the production of inflammatory cytokines, hyperactivation of B cells, excessive production of autoantibodies, and formation of immune complexes (ICs), which are key to the development of lupus nephritis (3–5). Follicular helper T cells (Tfhs), a subset of CD4^+^ T cells, participate in high-affinity B cell clone generation and long-lived memory in germinal centers (GCs) (6–8). They secrete large amounts of interleukin (IL)-21, which aids in GC formation, isotype switching, and plasma cell production (9). Tfhs play important roles in the production of antibodies and proinflammatory cytokines in SLE (10). Defective IL-2 production contributes to immune system imbalance in patients with SLE (11). Immunosuppressive and anti-inflammatory drugs are widely used to improve the symptoms of patients with SLE (12), but these therapies are not always effective. Among patients with SLE, 30−40% develop lupus nephritis (13–17). Despite adequate treatment, lupus nephritis may progress to end-stage renal disease, which can be fatal when patients do not undergo dialysis or kidney transplant (15, 18). In patients with lupus nephritis, the cumulative incidences of end-stage renal disease at 5, 10, and 15 years are 3–11%, 6–19%, and 19–25%, respectively (18).

Regulatory T cells (Tregs), a subset of CD4^+^ T cells, play a crucial role in the maintenance of immune tolerance by suppressing the aberrant immune responses of T and B cells (19). Humans and mice with mutations in the Treg lineage-determining transcription factor, Foxp3, do not have Tregs and display conventional T cell (Tconv) hyperreactivity, leading to lethal systemic autoimmunity. Reductions in the number of Tregs or defects in Treg function contribute to SLE pathogenesis (8, 9). Among Tregs, follicular Tregs (Tfrs) share features with Tfhs and conventional Tregs, and can inhibit Tfh and GC responses (10–12). The Tfh/Tfr ratio correlates with SLE disease activity (20). Selective enrichment of Tregs has been used to treat immune-mediated diseases (21, 22). Administration of low-dose IL-2, an essential cytokine for the maintenance and proliferation of Tregs, specifically expands Tregs *in vivo* (23) and is a promising approach for treating autoimmune diseases and graft versus host disease (GVHD) (24, 25). Short-term trials showed that low-dose IL-2 treatment promotes Treg production and inhibits T helper 17 cell and Tfh production (11, 26). The results of a randomized clinical trial suggested that low-dose IL-2 treatment is a beneficial and safe option for patients with SLE (27, 28).

In addition to IL-2, Tregs require T cell receptor (TCR) signaling for homeostasis and optimal proliferation (19, 29–31). However, Tregs, but not Tconvs, can proliferate in the absence of TCR stimulation following exogenous IL-2 administration (31). Our previous study showed that a combination of pharmacological TCR signaling inhibition and IL-2 administration can selectively expand Tregs, and this combination offered protection against experimental allergic encephalomyelitis (32). However, the combination of pharmacological calcineurin inhibition and IL-2 administration did not induce selective expansion of Tregs in a mouse model of acute GVHD (33). Thus, concomitant use of immunosuppressants should be carefully considered and tailored for therapeutic effect and effective Treg expansion induced by IL-2. It is unknown whether the combination treatment with IL-2 and a calcineurin inhibitor can selectively induce Treg expansion while inhibiting pathogenic Tfh expansion and exerting additive effects in SLE treatment. Therefore, in this study, we investigated the effects of the combination of IL-2 and a calcineurin inhibitor on Tregs and disease symptoms in a murine model of SLE-like chronic GVHD (cGVHD).

## 2 Materials and methods

### 2.1 Mice

Female *C57BL/6* (B6), (*C57BL/6* × *DBA/2*) F1 (BDF1), *DBA/2*, and *MRL/MpJmsSlc-lpr/lpr* (MRL/lpr) mice were purchased from SLC (Hamamatsu, Japan). Mice aged 7–9 weeks were used for the isolation of splenocytes or induction of GVHD. All mice were housed under specific pathogen-free conditions and were treated in strict compliance with the Animal Facility Regulations of Kansai Medical University. All animal studies were approved by the Animal Care Committee of Kansai Medical University (approval number 22-066).

### 2.2 Preparation of IL-2 complexes

The IL-2 IC was prepared by mixing 5 μg of anti-IL-2 antibody (clone JES6-1D; BioXCell, West Lebanon, NH, USA) with 1 μg of recombinant mouse IL-2 (PeproTech, Rocky Hill, NJ, USA), and incubating the mixture for 15 min on ice. After incubation, the volume of the mixture was adjusted to 200 µL using sterile phosphate-buffered saline (PBS). The IL-2 IC generated using the JES6-1D anti-IL-2 monoclonal antibody selectively stimulates Treg expansion and exhibits an immunosuppressive effect.

### 2.3 Flow cytometry, cell sorting, and data analysis

The following antibodies were used in flow cytometry: phycoerythrin (PE)-anti-CD25 (PC61), allophycocyanin (APC)-anti-cytotoxic T-lymphocyte-associated protein 4 (CTLA-4) (UC10-4F10-11), and Alexa Flour 647-anti-signal transducer and activator of transcription 5 (STAT5) (pY694, clone 47) from BD Pharmingen (San Diego, CA, USA); PE or APC-anti-Foxp3 (FJK-16s), phycoerythrin-Cy7 (PECy7)-anti-glucocorticoid-induced TNFR-related protein (GITR) (DTA-1), PECy7-anti-folate receptor 4 (FR4) (eBio12A5), APC-eFluor 780-anti-CD8α (53-6.7), APC-eFluor 780-anti-CD4 (RM4-5), Pacific Blue-anti-IL-4 (11B11), and SA-APC-eFluor 780 from eBioscience (San Diego, CA, USA); fluorescein isothiocyanate (FITC) or Pacific Blue-anti-TCRβ (H57-597), FITC-, peridinin chlorophyll protein Cy5.5 (PerCPCy5.5)- or Pacific Blue-anti-CD4 (RM4-5), FITC-anti-inducible T cell costimulator (ICOS) (C398.4A), FITC-anti-CXCR5 (L138D7), PE-anti-CD95 (Fas) (SA367H8), PerCPCy5.5-anti-H2-Kb (AF6-88.5), PerCPCy5.5-anti-B220 (RA3-6B2), PECy7-anti-CD62L (MEL-14), PECy7-anti-Bcl-2 (BCL/10C4), PECy7-anti-PD-1 (29F.1A12), APC-anti-IAb (AF6-120.1), biotin-anti-CD44 (IM7), biotin-anti-CD122 (5H4), biotin-anti-CXCR5 (L138D7), Pacific Blue-anti-CD8α (53-6.7), Pacific Blue-anti-Helios (22F6), Pacific Blue-anti-GL7 (GL7), biotin-anti-CD138 (281-2), Pacific Blue-anti-IFNγ (XMG1.2), APC-anti-IL-17A (TC11-18H10.1), Pacific Blue-anti-IL-10 (JES5-16E3), FcBlock (93), and Alexa Flour 647-anti-signal transducer and activator of transcription 3 (STAT3) phosphotyrosine antibody (13A3-1) from Biolegend (San Diego, CA, USA). An Aqua fluorescent live/dead cell stain kit was purchased from Invitrogen (Carlsbad, CA, USA). For phospho-flow analysis, freshly isolated spleen cells were fixed in 4% paraformaldehyde for 20 min at 4°C and permeabilized by incubation with 90% cold methanol for 30 min. The cells were stained with the antibody overnight at 4°C. To measure cytokine production, lymphocytes from the spleen were cultured in the presence of phorbol-12-myristate 13-acetate (50 ng/mL), ionomycin (1 μg/mL), and brefeldin A (10 μM) for 5 h before antibody staining. All analytical flow cytometry analyses were performed using a FACSCanto flow cytometry system (BD Biosciences, San Jose, CA, USA). The data were analyzed using FlowJo software (TreeStar, Ashland, OR, USA). Dead cells were excluded from analysis using a Live/Dead Fixable Aqua Dead Cell Stain kit (Invitrogen). Foxp3 is the most specific marker for distinguishing Tregs from other T cells (34). CD4^+^Foxp3^+^ T cells were defined as Tregs. Tfr and Tfh were defined based on CXCR5 and PD-1 staining. CXCR5^+^PD-1^+^ cells among CD4^+^TCRβ^+^Foxp3^+^ cells and CXCR5^+^PD-1^+^ cells among CD4^+^TCRβ^+^Foxp3^-^ cells were defined as Tfr and Tfh cells, respectively. For cell sorting, pan T cells were purified with T cell magnetic beads using MACS columns (Miltenyi Biotec, Bergisch Gladbach, Germany) before cell surface staining. Fluorescence activated cell sorting (FACS) was performed using a FACSAria cell sorter (BD Biosciences) at the Central Research Laboratory of Kansai Medical University.

### 2.4 Induction and assessment of GVHD

BDF1 mice were intraperitoneally administered with 60 × 10^6^ splenocytes, which were suspended in 200 μL sterile PBS, from DBA/2 or BDF1 mice. After splenocyte administration, the mice were monitored every 3 days, and proteinuria was determined every week. Proteinuria was assessed semi-quantitatively using Uropaper III (Eiken Chemical Co., Ltd., Tochigi, Japan). The urinary protein concentration was measured using a protein quantification assay (TaKaRa Bio, Shiga, Japan) according to the manufacturer’s instructions. Briefly, urine was diluted 1:10 and incubated for 5 min at room temperature (20°C–25°C) with Bradford dye reagent. After incubation, the absorbance of the samples at 595 nm was measured. The protein concentration was determined using a standard curve generated using dilutions of bovine serum albumin standard. PBS, tacrolimus (5 mg/kg), and IL-2 IC (200 μL) were intraperitoneally administered. Previous studies showed that 3-day IL-2 IC injection can induce nearly maximum expansion of Tregs (35, 36). Additionally, administration of IL-2 IC for at least four days worsened GVHD in our previous study (33). Thus, we performed injected IL-2 IC three times daily for 3 days. Tacrolimus was administered to wild-type mice for 3, 5, or 7 days. After injection, we observed comparable Treg reduction as that in non-injected mice (data not shown). We then administered tacrolimus for 7 days because the treatment duration of tacrolimus is typically long in clinical situations. In the prophylactic setting, host mice were treated with PBS (control), tacrolimus, IL-2 IC, or IL-2 IC plus tacrolimus starting on the day of GVHD induction. In the therapeutic setting, host mice were treated starting at 23 days after GVHD induction. Proteinuria scores were calculated using the following criteria in the MRL/lpr model: +2 was assigned 1 point, +3 was assigned 2 points, and +4 was assigned 3 points.

### 2.5 Immuno-histochemical analysis

The kidneys isolated from the host mice were embedded in OCT compound (Sakura Finetek, Alphen aan den Rijn, Netherlands) and snap-frozen on dry ice; sections (8 µm) were prepared on glass slides, fixed in acetone and ethanol for 5 min, and dried. After blocking with PBS containing 10% fetal calf serum for 30 min at room temperature, the sections were incubated with FITC-conjugated goat anti-mouse C3 antibody (MP Biomedicals, Irvine, CA, USA) diluted 1:500 in FACS buffer for 2 h at room temperature in the dark. To detect IC deposition, the sections were incubated with FITC-conjugated goat anti-mouse IgG antibody (Sigma Aldrich, St. Louis, MO, USA) diluted 1:320 in FACS buffer for 2 h at room temperature. The nucleus was stained using 4′,6-diamidino-2-phenylindole. Fluorescent staining was observed using a fluorescent microscope (Zeiss LSM700, Oberkochen, Germany). Fluorescence intensity was evaluated using ImageJ software (version 1.52a, National Institutes of Health, Bethesda, MD, USA).

### 2.6 Cytokine analysis

Peripheral blood was collected from the host mice at 2 days after transplantation. Cytokine concentrations in the serum were analyzed using the BD Cytometric Bead Array system (mouse Th1/Th2/Th17 cytokine kit; BD Biosciences) according to the manufacturer’s protocol.

### 2.7 ELISA

The anti-dsDNA IgG titer in the serum was measured using an anti-dsDNA IgG ELISA kit (Alpha Diagnostic International, San Antonio, TX, USA) following the manufacturer’s protocol.

### 2.8 Quantitative real-time polymerase chain reaction

T cells obtained from cGVHD mice at 28 days after cell transfer were sorted into CD4^+^CD25^+^ and CD4^+^CD25^-^ cells using a FACSAria cell sorter (BD Biosciences). Total RNA was purified from the FACS-sorted CD4^+^CD25^-^ cells using an RNeasy kit (Qiagen, Hilden, Germany). Polymerase chain reaction (PCR) and subsequent analyses were performed using a Corbett Rotorgene Q real-time cycler (Corbett Life Sciences, Sydney, Australia) with TaqMan Universal PCR master mix (Applied Biosystems, Foster City, CA, USA), primers (mouse IL-21: Mm00517640_m1; mouse GAPDH: Mm99999915_g1) (Thermo Fisher Scientific/Applied Biosystems), and labeled TaqMan probes (TaqMan Gene Expression Assays; Applied Biosystems). The relative amount of each mRNA was determined using the comparative CT method and was normalized to the level of *GAPDH* in each sample.

### 2.9 Statistical analysis

*t*-Tests for comparisons between groups and analysis of variance with Tukey’s *post-hoc* test for multiple-group comparisons were performed using Prism software (GraphPad, La Jolla, CA, USA) as appropriate. Results with *p* < 0.05 were considered as statistically significant.

## 3 Results

### 3.1 Combination of IL-2 and tacrolimus expands Tregs *in vivo*

**Figure 1:**
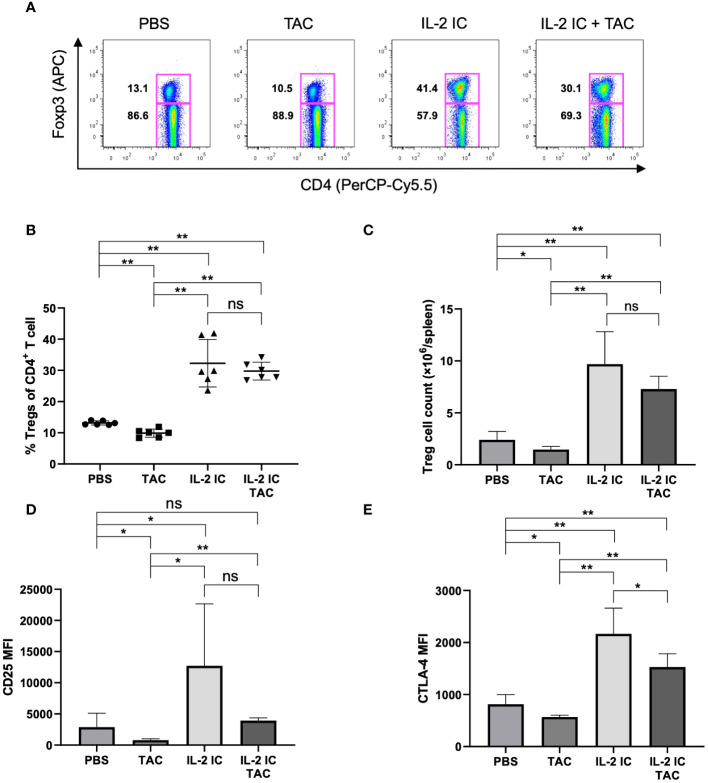
Effect of tacrolimus, interleukin-2 immune complexes (IL-2 IC), and IL-2 IC plus tacrolimus on the Treg proportion. B6 mice were intraperitoneally administered with vehicle (PBS), tacrolimus, IL-2 IC, or IL-2 IC plus tacrolimus for three consecutive days (0, 1, and 2), and T cells were analyzed using flow cytometry at day 4. **(A)** Representative fluorescence-activated cell sorting (FACS) plots. The plots were gated on live CD4^+^TCRβ^+^ cells. **(B)** Percentage and **(C)** absolute number of Tregs in splenocytes harvested on day 4. The mean fluorescence intensity of **(D)** CD25 and **(E)** CTLA-4. Compiled data from two independent experiments (n = 6 mice/group) are presented as the mean ± SD. *p < 0.05; **p < 0.001 calculated using unpaired, two-tailed Student’s *t*-test. ns, nonsignificant.

To investigate the effect of the combination of IL-2 and tacrolimus on Treg (CD4^+^Foxp3^+^T cell) production *in vivo*, wild-type B6 mice were administered a vehicle (PBS), tacrolimus, IL-2 IC, or IL-2 IC plus tacrolimus. The proportion and absolute number of Tregs were significantly reduced in mice administered tacrolimus alone compared with those in mice administered PBS; however, these parameters were significantly augmented in mice administered IL-2 IC or IL-2 IC plus tacrolimus compared with those in mice administered PBS (**Figures 1A–C**). The expression of CD25 and CTLA-4, which are commonly associated with Tregs, was significantly elevated in Tregs isolated from mice administered IL-2 IC compared with that in Tregs from mice administered PBS. The expression of Tregs isolated from mice administered tacrolimus was significantly reduced compared with that of Tregs from mice administered PBS (**Figures 1B, C**). The expression of CTLA-4, but not CD25, also increased in Tregs isolated from mice administered IL-2 IC plus tacrolimus compared with that of mice administered PBS (**Figures 1D, E**). These results indicate that augmentation of IL-2-induced Treg expression is maintained at a steady state, even if tacrolimus is used simultaneously.

### 3.2 Prophylactic administration of IL-2 IC, but not IL-2 IC plus tacrolimus, reduced the incidence of proteinuria

**Figure 2:**
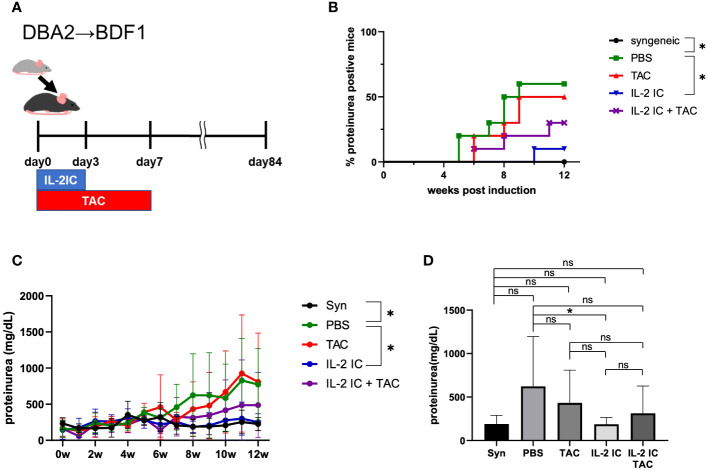
Prophylactic treatment of murine SLE-like chronic graft versus host disease. **(A)** Schematic representation of the treatment schedule. **(B)** Frequency of mice positive for proteinuria as determined from the elevated albumin level in the urine. **(C)** Changes in urine protein excretion levels. Compiled data from two independent experiments (n = 6−10 mice/group) are presented as the mean ± SD. *p < 0.05 calculated using ANOVA with Tukey’s *post-hoc* test. **(D)** Urine protein excretion levels at 8 weeks after induction of GVHD. Compiled data from two independent experiments (n = 6−10 mice/group) are presented as the mean ± SD. *p < 0.05 calculated using unpaired, two-tailed Student’s *t*-test. ns, nonsignificant.

We examined the prophylactic effect of IL-2 and tacrolimus on the development of SLE-like murine cGVHD (**Figure 2A**). The development of proteinuria was significantly reduced in host mice treated with IL-2 compared with that in control mice, as previously reported (37). However, the development of proteinuria in host mice treated with tacrolimus or IL-2 IC plus tacrolimus was not delayed (**Figure 2B**). The urine protein excretion in host mice treated with IL-2 IC was significantly reduced compared with that in control mice (**Figures 2C, D**). The urine protein excretion level in host mice treated with tacrolimus or IL-2 IC plus tacrolimus at 8 weeks after GVHD induction was reduced compared with that in control mice, but not significantly (**Figure 2D**). At 2 days after transplantation during the preclinical phase, the concentrations of serum proinflammatory cytokines, such as TNF-α, IFN-γ, and IL-2, were higher in host mice treated with IL-2 IC and IL-2 IC plus tacrolimus than those in control mice (**Supplementary Figure S1A**). Although serum anti-dsDNA antibody titers in mice treated with tacrolimus or IL-2 IC plus tacrolimus were decreased relative to those in control mice, those in mice treated with IL-2 IC were greatly decreased (**Supplementary Figure S1B**). These results suggest that prophylactic treatment with a combination of IL-2 IC and tacrolimus does not have an additive effect on proteinuria in SLE-like GVHD, whereas IL-2 ameliorates proteinuria.

### 3.3 Prophylactic administration of IL-2 IC plus tacrolimus expands Tregs while inhibiting expansion of Tfhs in cGVHD mice

**Figure 3:**
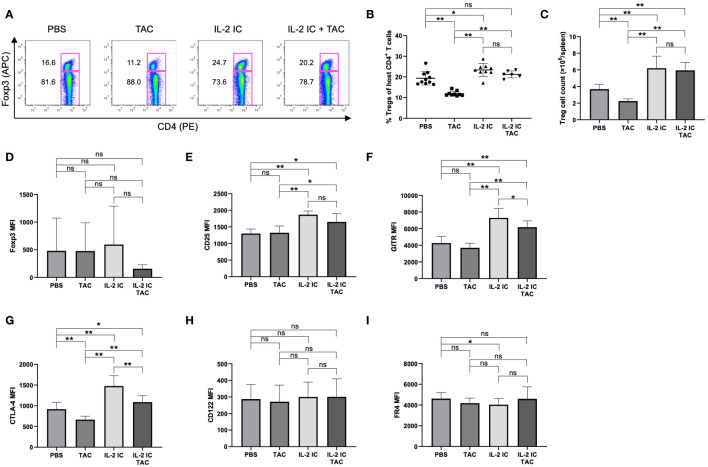
Regulatory T cells (Tregs) in host splenocytes from chronic graft versus host disease mice prophylactically treated with vehicle (PBS), tacrolimus, interleukin-2 immune complexes (IL-2 IC), or IL-2 IC plus tacrolimus. **(A)** Representative fluorescence-activated cell sorting (FACS) plots of host-derived CD4^+^T cells. The plots were gated on live H-2Kb^-^TCRβ^+^CD4^+^ cells. **(B)** Percentage and **(C)** absolute number of host-derived Tregs in splenocytes harvested on day 7. Compiled data from two independent experiments (n = 6−10 mice/group) are presented as the mean ± SD. Host-derived Tregs were analyzed for expression of **(D)** Foxp3, **(E)** CD25, **(F)** GITR, **(G)** CTLA-4, **(H)** CD122, and **(I)** FR4. Compiled mean fluorescence intensity data from five independent experiments (n = 9−13 mice/group) are presented as the mean ± SD. *p < 0.05; **p < 0.001 calculated using unpaired, two-tailed Student’s *t*-test. IL-2 IC, interleukin-2 immune complexes; ns, nonsignificant.

**Figure 4:**
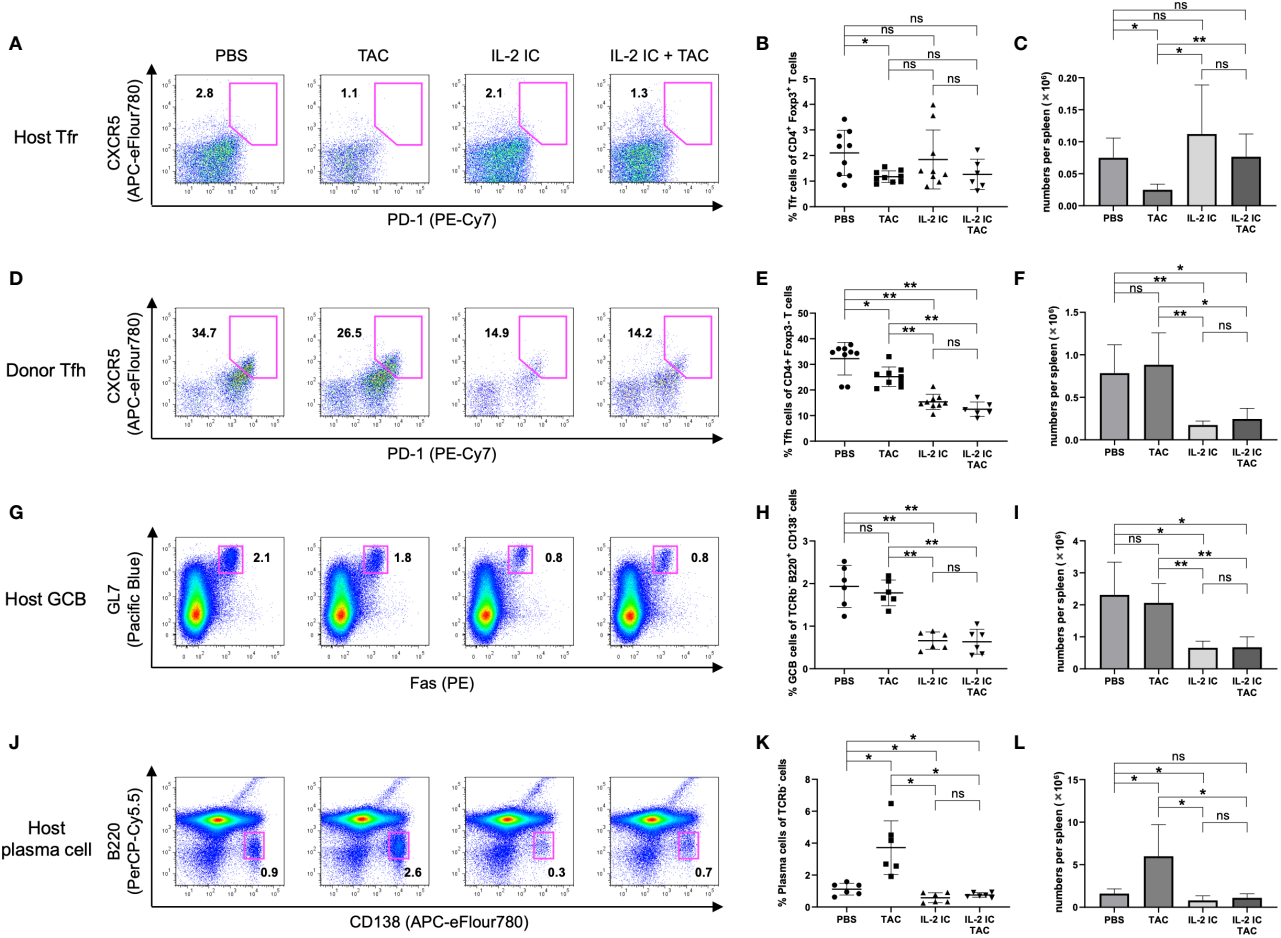
Follicular regulatory T (Tfr), follicular helper T (Tfh), germinal center B (GCB), and plasma cells in splenocytes harvested on day 7 after transplantation from chronic graft versus host disease mice prophylactically treated with vehicle (PBS), tacrolimus, IL-2 IC, or IL-2 IC plus tacrolimus. **(A)** Representative fluorescence-activated cell sorting (FACS) plots of host-derived Tfrs (CD4^+^CXCR5^+^PD-1^+^Foxp3^+^). The plots were gated on live CD4^+^TCRβ^+^Foxp3^+^ cells. **(B)** Proportion and **(C)** absolute number of host-derived Tfrs. **(D)** Representative FACS plots of donor-derived Tfhs (CD4^+^CXCR5^+^PD-1^+^Foxp3^–^). The plots were gated on live CD4^+^TCRβ^+^Foxp3^-^ cells. **(E)** Proportion and **(F)** absolute number of donor-derived Tfhs. **(G)** Representative FACS plots of host-derived GCBs (TCRβ^–^ B220^+^CD138^–^GL-7^+^Fas^+^). The plots were gated on live TCRβ^-^B220^+^CD138^-^ cells. **(H)** Proportion and **(I)** absolute number of host-derived GCBs. **(J)** Representative FACS plots of host-derived plasma cells (TCRβ^–^B220^–^CD138^+^). The plots were gated on live TCRβ^-^ cells. **(K)** Proportion and **(L)** absolute number of host-derived plasma cells. Compiled data from three independent experiments are presented as the mean ± SD; n = 6−9 mice/group. *p < 0.05, **p < 0.001 calculated using unpaired, two-tailed Student’s *t*-test. IL-2 IC, interleukin-2 immune complexes; ns, nonsignificant.

We investigated whether a combination of IL-2 and tacrolimus expanded host-derived Tregs in a prophylactic setting. The proportion and absolute number of Tregs were significantly increased in host mice treated with IL-2 IC compared with those in control mice, whereas these values were significantly decreased in host mice treated with tacrolimus (**Figures 3A–C**). The proportion of Tregs in host mice treated with IL-2 IC plus tacrolimus did not increase compared with that in control mice; however, there was a significant increase compared with that in mice treated with tacrolimus alone (**Figures 3A, B**). The absolute number of Tregs in host mice treated with IL-2 IC was significantly higher than that in control mice (**Figure 3C**). The absolute number of Tregs in host mice treated with IL-2 IC plus tacrolimus was significantly higher than that in control or host mice treated with tacrolimus (**Figure 3C**). Additionally, we analyzed surface markers commonly associated with Tregs (**Figures 3D–I**). Tregs isolated from host mice treated with IL-2 IC or IL-2 IC plus tacrolimus expressed significantly higher levels of CD25, GITR, and CTLA-4 than those from control mice, whereas the expression of Foxp3, CD122, and FR4 in host mice treated with IL-2 IC or IL-2 IC plus tacrolimus was comparable to that in control mice. Tregs isolated from host mice treated with IL-2 expressed significantly lower levels of FR4 than those isolated from control mice. Furthermore, Tregs isolated from host mice treated with tacrolimus showed significantly lower levels of CTLA-4 than those from control mice. Tfrs are a Treg subset sharing features with Tfhs and conventional Tregs that can inhibit Tfh and GC responses. The proportion of Tfrs in host mice treated with IL-2 IC or IL-2 IC plus tacrolimus was comparable to that in control mice, whereas the proportion in host mice treated with tacrolimus alone significantly decreased compared with that in control mice (**Figures 4A, B**, **Supplementary Figure S2**). The absolute number of Tfrs in host mice treated with IL-2 IC or IL-2 IC plus tacrolimus was comparable to that in control mice (**Figure 4C**). The absolute number of Tfrs in host mice treated with tacrolimus was significantly lower than that in control mice, and the absolute number of Tfrs in host mice treated with IL-2 IC plus tacrolimus was significantly higher than that in host mice treated with tacrolimus alone (**Figure 4C**). The proportion and absolute number of Tfhs were significantly decreased in host mice treated with IL-2 IC compared with those in control mice (**Figures 4D–F**). In addition, the proportion and absolute number of Tfhs in host mice treated with IL-2 IC plus tacrolimus were significantly decreased compared with those in control mice (**Figures 4D, F**). The Tfr/Tfh ratio was increased in host mice treated with IL-2 IC or IL-2 IC plus tacrolimus compared with that in control mice, whereas the ratio in host mice treated with tacrolimus was decreased (**Supplementary Figure S3**). Effector memory T cell (T_EM_; CD4^+^ CD44^+^ CD62L^-^) and central memory T cell (T_CM_; CD4^+^CD44^+^CD62L^+^) proportions in host mice treated with IL-2 IC plus tacrolimus were decreased compared with those in control mice (**Supplementary Figure S4**). The proportion and absolute number of GC B cells (GCB) (B220^+^CD138^–^GL-7^+^Fas^+^) and plasma cells were significantly decreased in host mice treated with IL-2 IC compared with those in control mice (**Figures 4G–L**). The proportion and absolute number of GCBs in host mice treated with tacrolimus did not decrease (**Figures 4G–I**). The proportion and absolute number of plasma cells in host mice treated with tacrolimus were higher than those in control mice (**Figures 4J–K**). In host mice treated with IL-2 IC plus tacrolimus, the proportion and absolute number of GCBs were significantly decreased compared with those in control mice (**Figures 4G–I**); in addition, the proportion of plasma cells was significantly decreased compared with that in control mice (**Figures 4J, K**). The absolute number of plasma cells was comparable to that in the control (**Figures 4L**). These results suggest that IL-2-treatment can increase Treg proportions with or without tacrolimus while inhibiting the production of detrimental lymphocyte subsets in the development of cGVHD in a prophylactic setting.

### 3.4 Therapeutic administration of IL-2 IC plus tacrolimus (but not IL-2 IC) protects against proteinuria in cGVHD

**Figure 5:**
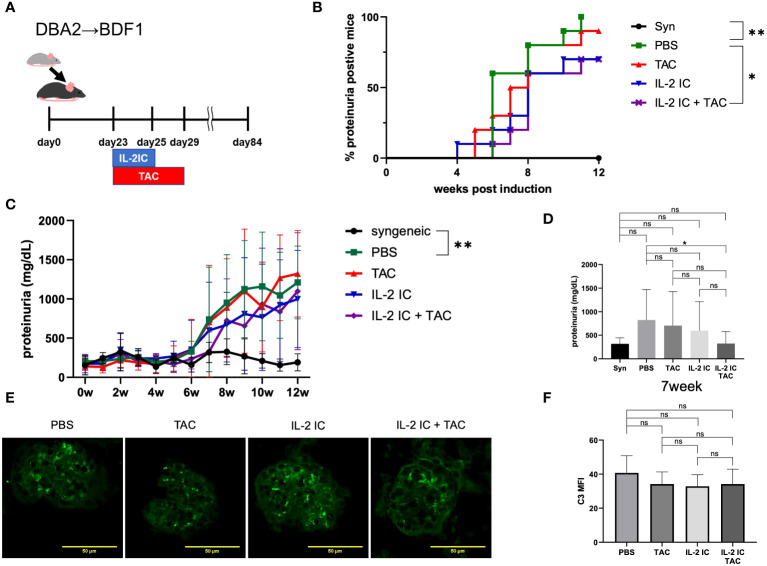
Therapeutic treatment of murine SLE-like chronic graft versus host disease. **(A)** Schematic representation of the treatment schedule. **(B)** Frequency of mice positive for proteinuria as determined from the elevated albumin level in the urine, and **(C)** changes in urine protein excretion levels. Compiled data from two independent experiments are presented as the mean ± SD; n = 6−10 mice/group. *p < 0.05 calculated using ANOVA with Tukey’s post-hoc test. **(D)** Urine protein excretion levels at 7 weeks after induction of GVHD. Data from two independent experiments are presented as the mean ± SD; n = 6−10 mice/group. **(E)** Immuno-histochemical staining of C3 deposition in the glomeruli of host mice. Kidneys were obtained from host mice at 30 days after *DBA/2* mouse splenocyte transplantation. Cryosections were prepared and stained for C3 deposition. **(F)** Mean fluorescence intensity data from two independent experiments are presented as the mean ± SD; n = 5−6 mice/group. *p < 0.05; **p < 0.001 calculated using unpaired, two-tailed Student’s *t*-test. ns: nonsignificant.

We investigated the therapeutic effect of IL-2 plus tacrolimus against proteinuria in SLE-like murine cGVHD (**Figure 5A**). Therapeutic administration of tacrolimus and IL-2 IC slightly delayed the development of proteinuria. Protein levels in the urine of host mice treated with IL-2 IC were slightly decreased compared with those in the urine of control mice, which was not observed in host mice treated with tacrolimus (**Figures 5B–D**). The development of proteinuria in host mice treated with IL-2 IC plus tacrolimus was significantly slower than that in control mice. The urine protein excretion level at 7 weeks after cGVHD induction was significantly reduced in host mice treated with IL-2 IC plus tacrolimus compared with that in control mice, whereas there was no significant reduction in urine protein excretion in host mice treated with tacrolimus or IL-2 IC (**Figure 5D**). Immuno-histochemical analysis of the kidneys of host mice showed marginally decreased renal deposition of complement and ICs compared with the levels in the kidneys of control mice, regardless of treatment (**Figures 5E, F**, **Supplementary Figure S5**). In addition, the therapeutic efficacy of IL-2 IC plus tacrolimus was observed in MRL/lpr mice, a lupus model (**Supplementary Figure S6**), indicating that the beneficial effect of IL-2 IC concomitantly used with tacrolimus is not restricted to SLE-like GVHD models. These results suggest that administration of IL-2 IC plus tacrolimus was therapeutically effective against proteinuria in SLE-like murine cGVHD, although short-term tacrolimus or IL-2 IC treatment alone was not effective.

### 3.5 Therapeutic administration of IL-2 IC plus tacrolimus effectively inhibits expansion of Tfhs in cGVHD mice

**Figure 6:**
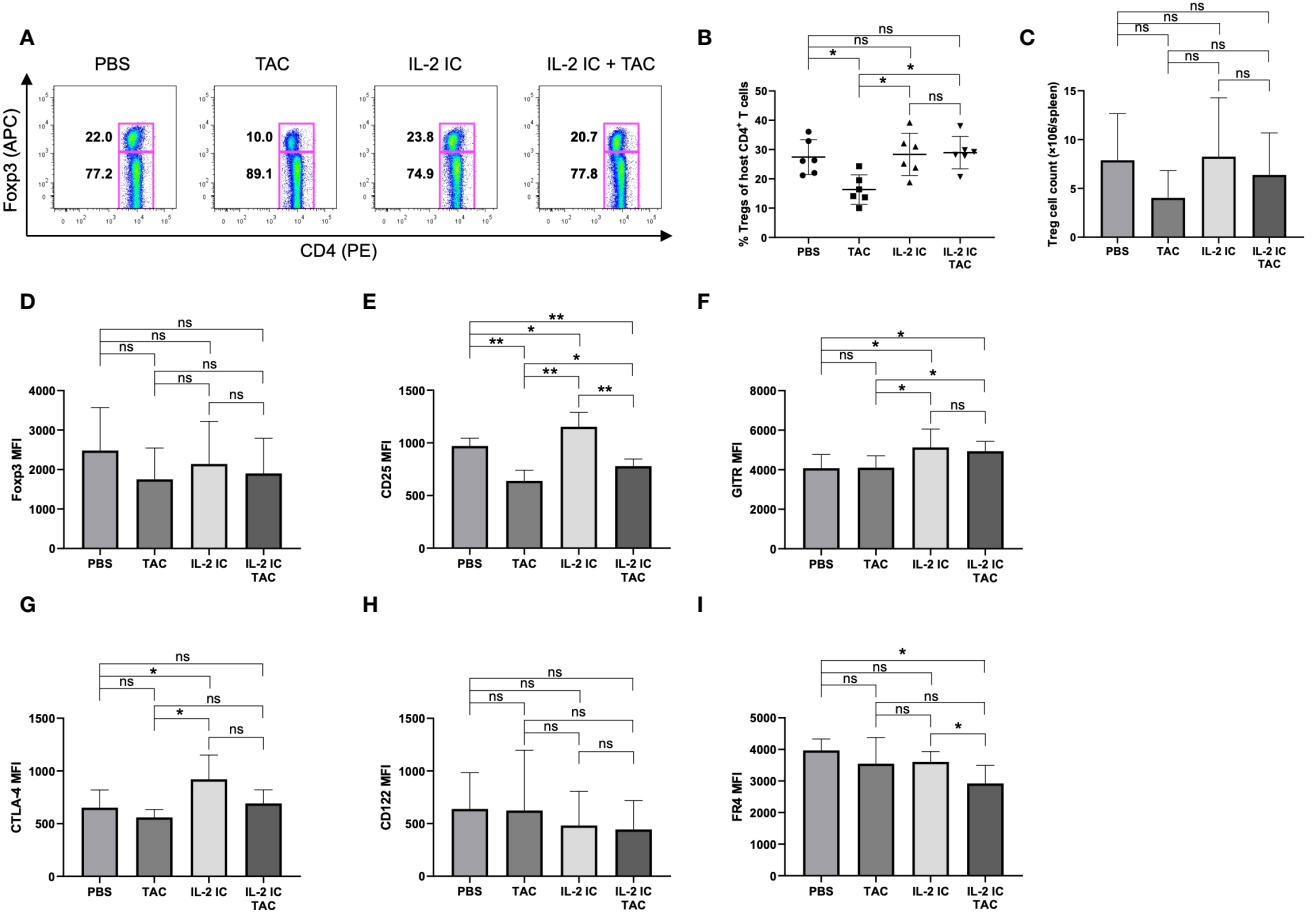
Regulatory T cells (Tregs) in host splenocytes harvested on day 30 after transplantation from chronic graft versus host disease mice therapeutically treated with vehicle (PBS), tacrolimus, interleukin-2 immune complexes (IL-2 IC), or IL-2 IC plus tacrolimus. **(A)** Representative fluorescence-activated cell sorting (FACS) plots of host-derived CD4^+^ T cells. The plots were gated on live H-2Kb^-^TCRβ^+^CD4^+^ cells. **(B)** Percentage and **(C)** absolute number of host-derived Tregs in splenocytes harvested on day 30. Data from two independent experiments are presented as the mean ± SD; n = 6 mice/group. Host-derived Tregs were analyzed for the expression of **(D)** Foxp3, **(E)** CD25, **(F)** GITR, **(G)** CTLA-4, **(H)** CD122, and **(I)** FR4. The mean fluorescence intensity data from two independent experiments are presented as the mean ± SD; n = 6 mice/group. *p < 0.05; **p < 0.001 calculated using unpaired, two-tailed Student’s *t*-test. ns, nonsignificant.

We investigated whether therapeutic administration of IL-2 IC plus tacrolimus expands host-derived Tregs and Tfrs, which can inhibit the detrimental expansion of Tfhs, GCBs, and plasma cells in cGVHD. The proportion and absolute number of host-derived Tregs in host mice treated with IL-2 IC plus tacrolimus were comparable to those in control or host mice treated with IL-2 IC, whereas those of Tregs were significantly decreased in host mice treated with tacrolimus compared with the levels in control mice (**Figures 6A–C**). The proportion of Tregs in host mice treated with IL-2 IC plus tacrolimus was significantly higher than that in host mice treated with tacrolimus, indicating that addition of IL-2 can restore Treg proportions even when tacrolimus is administered. The expression of Foxp3 and CD122 was comparable among all groups (**Figures 6D, G**). Tregs isolated from host mice treated with IL-2 expressed significantly higher levels of CD25, GITR, and CTLA-4 than those from control mice (**Figures 6E, F, H**). Furthermore, Tregs isolated from host mice treated with tacrolimus or IL-2 IC plus tacrolimus expressed a significantly lower level of CD25 than those from control mice, whereas Tregs from these control mice also had a significantly higher level of CD25 compared with Tregs from host mice treated with tacrolimus (**Figure 6E**). Tregs isolated from host mice treated with IL-2 IC plus tacrolimus expressed significantly higher levels of GITR than those from control mice; however, the expression of FR4 was significantly lower than that in control mice (**Figures 6F, I**). The Tfr/Tfh ratio remained constant among host groups (**Supplementary Figure S7**). The number of T_EM_ in host mice treated with IL-2 IC plus tacrolimus decreased compared with that in control mice, whereas the number of T_CM_ cells remained unaltered (**Supplementary Figure S8**).

**Figure 7:**
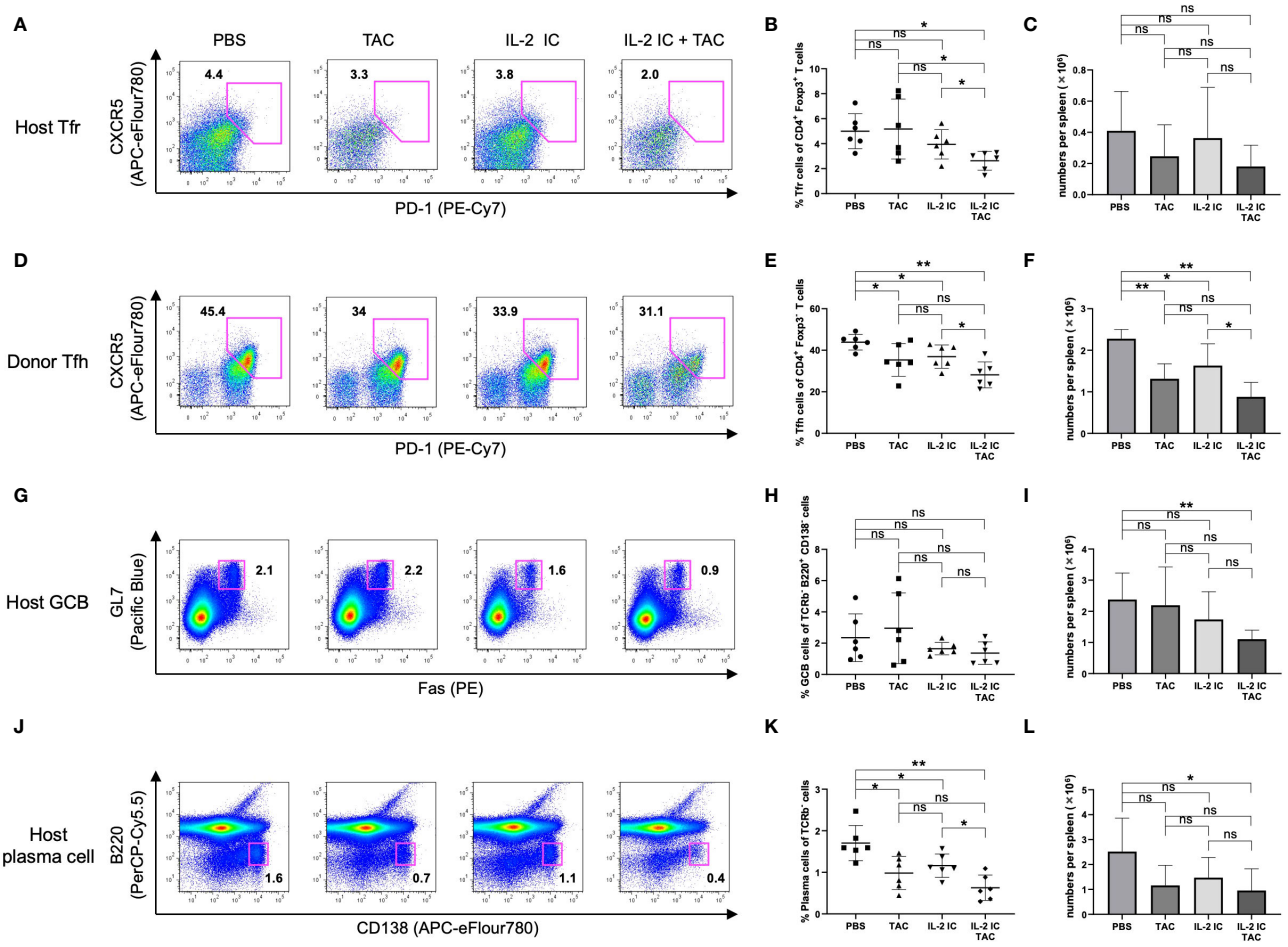
Follicular regulatory T (Tfr), follicular helper T (Tfh), germinal center B (GCB), and plasma cells in splenocytes harvested on day 30 after transplantation from chronic graft versus host disease mice therapeutically treated with vehicle (PBS), tacrolimus, IL-2 IC, or IL-2 IC plus tacrolimus. **(A)** Representative FACS plots of host-derived Tfrs (CD4^+^CXCR5^+^PD-1^+^Foxp3^+^). The plots were gated on live CD4^+^TCRβ^+^Foxp3^+^ cells. **(B)** Proportion and **(C)** absolute number of host-derived Tfrs. **(D)** Representative FACS plots of donor-derived Tfhs (CD4^+^CXCR5^+^PD-1^+^Foxp3^-^). The plots were gated on live CD4^+^TCRβ^+^Foxp3^-^ cells. **(E)** Proportion and **(F)** absolute number of donor-derived Tfhs. **(G)** Representative FACS plots of host-derived GCBs (TCRβ^-^B220^+^CD138^-^GL-7^+^Fas^+^). The plots were gated on live TCRβ^-^B220^+^CD138^-^ cells. **(H)** Proportion and **(I)** absolute number of host-derived GCBs. **(J)** Representative FACS plots of host-derived plasma cells (TCRβ^-^B220^-^CD138^+^). The plots were gated on live TCRβ^-^ cells. **(K)** Proportion and **(L)** absolute number of host-derived plasma cells. Compiled data from two independent experiments are presented as the mean ± SD; n = 6 mice/group. *p < 0.05, **p < 0.001 calculated using unpaired, two-tailed Student’s *t*-test. ns, nonsignificant.

Unexpectedly, the proportion of Tfrs in host mice treated with IL-2 IC plus tacrolimus was significantly decreased compared with that in control mice (**Figures 7A, B**). The absolute number of Tfrs was comparable among groups (**Figure 7C**). The proportion and absolute number of Tfhs significantly decreased in host mice treated with IL-2 IC compared with those in control mice (**Figures 7D, E**). The proportion and absolute number of Tfhs in host mice treated with IL-2 IC plus tacrolimus were significantly decreased compared with those in control mice, whereas these parameters in host mice treated with tacrolimus and IL-2 IC were decreased to a lesser extent (**Figures 7D–F**). The Tfr/Tfh ratio remained unaltered in host mice treated with tacrolimus, IL-2 IC, or IL-2 IC plus tacrolimus compared with that in control mice (**Supplementary Figure S7**). The number of T_EM_ cells in host mice treated with IL-2 plus tacrolimus was significantly decreased compared with that in control mice, whereas the number of T_CM_ cells remained unaltered (**Supplementary Figure S8**). The proportion of GCBs was comparable among groups (**Figures 7G, H**). The absolute number of GCBs in host mice treated with IL-2 IC plus tacrolimus was significantly reduced compared with that in control mice (**Figure 7I**). The proportion of plasma cells in host mice treated with tacrolimus, IL-2 IC, and IL-2 IC plus tacrolimus was significantly lower than that in control mice, whereas the proportion in host mice treated with tacrolimus and IL-2 IC was decreased to a lesser extent (**Figures 7J, K**). The absolute number of cells in host mice treated with IL-2 IC plus tacrolimus was significantly lower than that in control mice (**Figure 7L**). These results suggest that a combination of IL-2 IC and tacrolimus can maintain Treg proportions and inhibit the production of detrimental lymphocyte subsets in cGVHD in the therapeutic setting.

### 3.6 Therapeutic administration of IL-2 IC plus tacrolimus decreases phosphorylation of STAT3, which is a positive regulator of Tfhs

**Figure 8:**
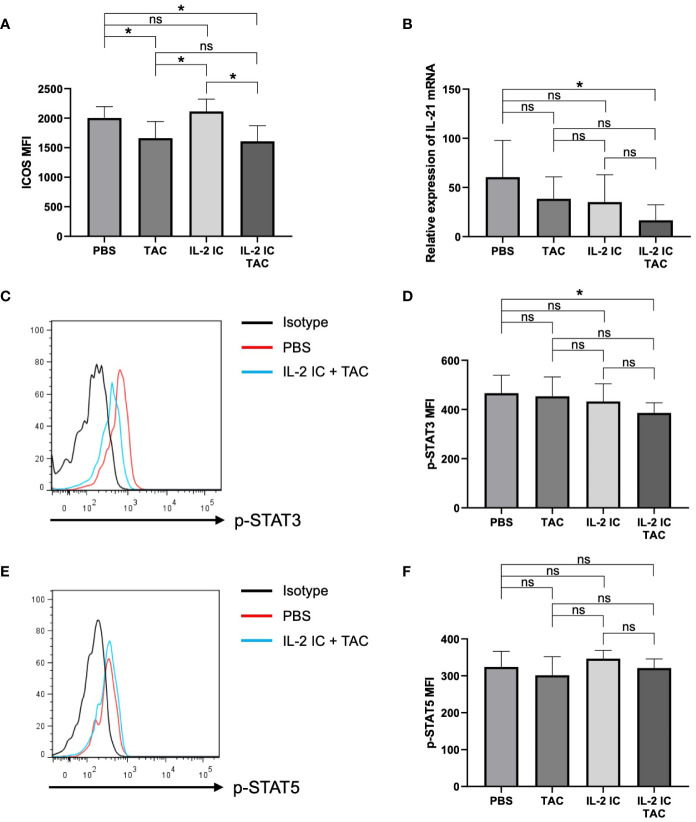
Effect of therapeutic administration on follicular helper T cells (Tfhs). T cells in splenocytes harvested on day 30 after transplantation from chronic graft versus host disease mice therapeutically treated with vehicle (PBS), tacrolimus, interleukin-2 immune complexes (IL-2 IC), or IL-2 IC plus tacrolimus were analyzed. **(A)** Mean fluorescence intensity of ICOS on Tfhs. Data from two independent experiments are presented as the mean ± SD; n = 6 mice/group. **(B)** Expression of *IL-21* mRNA in CD4^+^ T cells analyzed using polymerase chain reaction. *IL-21* mRNA level in GVHD host mice compared to that in wild type B6 mice. Data from two independent experiments are presented as the mean ± SD; n = 6−7 mice/group. **(C–F)** Level of phosphorylated STAT3 and STAT5 in Tfhs of chronic graft versus host disease mice was quantified using phospho-flow analysis. **(C)** Representative histogram and **(D)** mean fluorescence intensity of phosphorylated STAT3. **(E)** Representative histogram and **(F)** mean fluorescence intensity of phosphorylated STAT5. Compiled data from two independent experiments are presented as the mean ± SD; n = 6−7 mice/group. *p < 0.05 calculated using unpaired, two-tailed Student’s *t*-test. ns, nonsignificant.

To precisely examine the effect of the combination of IL-2 and tacrolimus on Tfhs, we evaluated ICOS expression, IL-21 synthesis, and STAT3 and STAT5 phosphorylation in the therapeutic setting; these factors are associated with the regulation and function of Tfhs. The expression of ICOS in host mice treated with tacrolimus or IL-2 IC plus tacrolimus was significantly lower than that in control mice (**Figure 8A**). Although serum IL-21 was not detected, the *IL-21* mRNA level in CD4^+^ T cells from host mice treated with IL-2 IC plus tacrolimus was significantly reduced compared with that in control mice, whereas the level in host mice treated with tacrolimus or IL-2 IC remained comparable to that in control mice (**Figure 8B**). STAT3 is a critical positive regulator of Tfh differentiation, and STAT3 deficiency is associated with a reduced circulating Tfh proportion, defective IL-21 expression, and B helper activity (38, 39). Phosphorylation of STAT3 in Tfhs isolated from host mice treated with IL-2 IC plus tacrolimus significantly reduced compared with that in Tfhs isolated from the control mice, whereas it remained constant in host mice treated with tacrolimus or IL-2 IC (**Figures 8C, D**). Phosphorylation of STAT5, a negative regulator of Tfh generation (40, 41), was comparable among groups (**Figures 8E, F**). Collectively, these results suggest that IL-2 plus tacrolimus induced a therapeutic effect in mice with SLE-like cGVHD via modulation of the positive regulator, and not the negative regulator, of Tfhs.

### 3.7 Therapeutic administration of IL-2 IC plus tacrolimus changes the balance of cytokine-producing CD4^+^ T cells

To explore the effect of therapeutic administration of IL-2 plus tacrolimus for cytokine expression of T cells, we analyzed T cells using flow cytometry on day 35 after administration of splenocytes (**Supplementary Figure S9**). The proportions of INF-γ^+^ and IL-4^+^CD4^+^ T cells were comparable among groups. The proportions of IL-17^+^ and IL-10^+^CD4^+^ T cells were significantly increased in host mice treated with IL-2 IC plus tacrolimus compared with those in control mice, whereas those in mice treated with tacrolimus or IL-2 IC were not changed.

## 4 Discussion

We investigated whether combination treatment with a calcineurin inhibitor (tacrolimus) and IL-2 can selectively induce Treg expansion while inhibiting Tfh expansion, exerting an additive treatment effect against lupus nephritis-like symptoms. We demonstrated that prophylactic use of the combination of a calcineurin inhibitor and IL-2 induces Treg expansion, and therapeutic use of this combination can maintain Treg proportions and ameliorate proteinuria in a murine model of SLE-like cGVHD. Pharmacological inhibition of calcineurin by tacrolimus negatively affected the Treg proportion; however, the addition of IL-2 restored the Treg proportion and led to a reduction in Tfh, GCB, and plasma cell proportions in the therapeutic setting. Our results suggest that the combination of IL-2 and a calcineurin inhibitor exerts an additive effect in the treatment of lupus nephritis.

Autoimmune disease-prone mice, which spontaneously develop lupus-like diseases, are commonly used to investigate SLE. The cGVHD mouse model is often used to investigate SLE-like diseases. In addition, cGVHD, which is induced in (*C57BL/6* × *DBA/2*) F1 (BDF1) mice by injecting *DBA/2* spleen cells, is associated with the activation of donor CD4^+^ T cells that recognize host major histocompatibility complex antigens and drive host B cell hyperactivity (42, 43). These host mice develop symptoms that resemble those of SLE, including high titers of anti-nuclear antibodies and anti-isologous erythrocyte (anti-red blood cell) antibodies and fatal IC-mediated glomerulonephritis (44, 45). This model enabled us to manipulate disease development and facilitate therapeutic intervention and investigation of pathological immune cells.

Tregs require IL-2 and adequate stimulation of TCR signaling for homeostasis and optimal proliferation (19, 29–31). However, Tregs can proliferate in the absence of adequate TCR stimulation when exogenous IL-2 is administered (31). Stimulation of TCR signaling is indispensable for the expansion of Tconvs. This differential dependency of Tregs and Tconvs on TCR signaling supports the beneficial effect of IL-2 administration plus calcineurin inhibition in diverse immune diseases. Therefore, treatment with the combination of IL-2 and a calcineurin inhibitor can induce Treg expansion while inhibiting the expansion of inflammatory cytokine-producing CD4^+^ T cells, resulting in an additive effect in experimental autoimmune encephalomyelitis (32). However, this combination could not induce these beneficial effects in acute GVHD (33), suggesting that this additive effect would not be obtained for alloimmunity and under strong inflammatory conditions, such as acute GVHD. The former possibility may not be likely because the therapeutic effect of the combination of IL-2 and tacrolimus was observed against SLE-like cGVHD in this study.

Tfrs are a Treg subset mainly localized in GCs; they can inhibit IL-21 and IL-4 expression by Tfh cells (46, 47). These cells also control the activity of GCBs and suppress the initial activation of B cells via epigenetic changes and multiple pathways (47–49). Imbalanced Tfr/Tfh ratios and the frequency of Tfr cells are correlated with the disease activity index and serum autoantibody levels (47, 50). In this study, therapeutic use of the combination of tacrolimus and IL-2 maintained the Treg proportions, whereas prophylactic use increased the proportions of Tregs and Tfrs. Tregs may contribute to the attenuation of proteinuria in the therapeutic setting because they also regulate Tfhs and humoral immunity by downregulating the expression of the costimulatory molecules B7-1 and B7-2 through CTLA-4 (51, 52).

According to a previous study (37), IL-2 treatment did not attenuate proteinuria in a therapeutic setting; however, we found that prophylactic IL-2 treatment attenuated proteinuria. A recent study suggested that exogenous IL-2 restores the function of Tfrs by converting Tfhs to Tfrs in patients with SLE (53). Alternatively, IL-2 potently suppresses the expression of Tfhs, primarily by inhibiting BCL6, which is crucial for primed CD4^+^ T cells to commit to Tfh differentiation (54, 55). Therefore, IL-2 therapy may inhibit early differentiation of Tfhs rather than destroy established Tfhs. Consistent with this finding, IL-2 treatment substantially decreased the Tfh proportion in the prophylactic setting, but marginally decreased the Tfh proportion in the therapeutic setting. Noticeable expansion of Tregs and Tfrs did not occur with the combination treatment of IL-2 and tacrolimus in the therapeutic setting. In addition to the marginal inhibition of Tfh development by IL-2, inhibition of T cell activation, which was induced by tacrolimus, may have contributed to the decreased proportion of Tfhs.

An imbalance in CD4^+^ T cell subsets is considered as an important factor underlying the pathology of SLE. IL-17 may be associated with disease activity and SLE etiology (56). In the current study, IL-17^+^ T cells increased in host mice treated with IL-2 plus tacrolimus; however, the proportion of T cells expressing IFN-γ, a positive regulator of Tfh differentiation (57), was not changed. IL-10 may exert differential effects on autoantibody development when it is applied before disease onset or afterward. *In vivo* blocking of IL-10 starting after disease onset increases the production of autoantibodies and lupus pathogenesis (58). Additionally, continuous blockade of IL-10 from birth delays the onset of autoantibody production and disease symptoms, such as proteinuria and glomerulonephritis (59). In this context, the increase in IL-10^+^ T cells in mice treated with IL-2 plus tacrolimus may have contributed to the inhibition of disease activity in the present study.

This study had some limitations. The combination of IL-2 and tacrolimus efficiently ameliorated proteinuria in the therapeutic setting, whereas addition of tacrolimus to IL-2 was not effective in the prophylactic setting. The prophylactic setting is not clinically relevant in autoimmune diseases, and the precise mechanism could not be determined. Moreover, we did not observe significant improvement in the deposition of complement and ICs in mice treated with the combination of IL-2 and tacrolimus in the therapeutic setting. Following the renal deposition of ICs and complement, their clearance may be slow, even if the mice are treated. We could not directly investigate the function of Tregs. Calcineurin inhibitors interfere with Treg suppressive functions (60). However, the expression of coreceptors commonly associated with Treg function in mice treated with IL-2 and tacrolimus, except for CD25 and FR4, did not decrease compared with that in control mice. The level of GITR was elevated by IL-2, with or without tacrolimus, compared with that in control mice. Addition of IL-2 to tacrolimus may maintain Treg function at a level similar to that of Tregs in mice treated with PBS.

In conclusion, pharmacological TCR inhibition and IL-2 decreased the Tfh proportion in a murine model of SLE-like cGVHD, and therapeutic use of a combination of tacrolimus and IL-2 efficiently ameliorated proteinuria. The regulation of Tfh production using a combination of a calcineurin inhibitor and IL-2 may be a logically appropriate therapeutic strategy for lupus nephritis. Clinical studies are necessary to confirm our findings and determine whether the proposed strategy can be applied for the treatment of SLE in humans.

## Data availability statement

The raw data supporting the conclusions of this article will be made available by the authors, without undue reservation.

## Ethics statement

The animal study was approved by Animal Care Committee of Kansai Medical University. The study was conducted in accordance with the local legislation and institutional requirements.

## Author contributions

YN: Writing – original draft, Investigation, Formal analysis. AS: Writing – review & editing, Writing – original draft, Supervision, Project administration, Data curation, Conceptualization. RT: Writing – review & editing, Investigation. RS: Writing – review & editing, Investigation. YT: Writing – review & editing, Investigation. HY: Writing – review & editing, Investigation. TI: Writing – review & editing, Supervision.
