# Supplementary material for: Concomitant use of interleukin-2 and tacrolimus suppresses follicular helper T cell proportion and exerts therapeutic effect against lupus nephritis in systemic lupus erythematosus-like chronic graft versus host disease
Source: Front Immunol. 2024 Apr 11;15:1326066. doi: 10.3389/fimmu.2024.1326066 (PMC11043470; doi:10.3389/fimmu.2024.1326066)
Supplement: Supplementary file 1 [file DataSheet_1.docx]

Supplementary Material

**Concomitant use of interleukin-2 and tacrolimus suppresses follicular helper T cell levels and exerts therapeutic effect on lupus nephritis in systemic lupus erythematosus-like chronic graft versus host disease**

Yutaro Nasa, Atsushi Satake*, Ryohei Tsuji, Ryo Saito, Yukie Tsubokura, Hdeaki Yoshimura, Tomoki Ito

*** Correspondence:** Atsushi Satake: satake_at@yahoo.co.jp

#
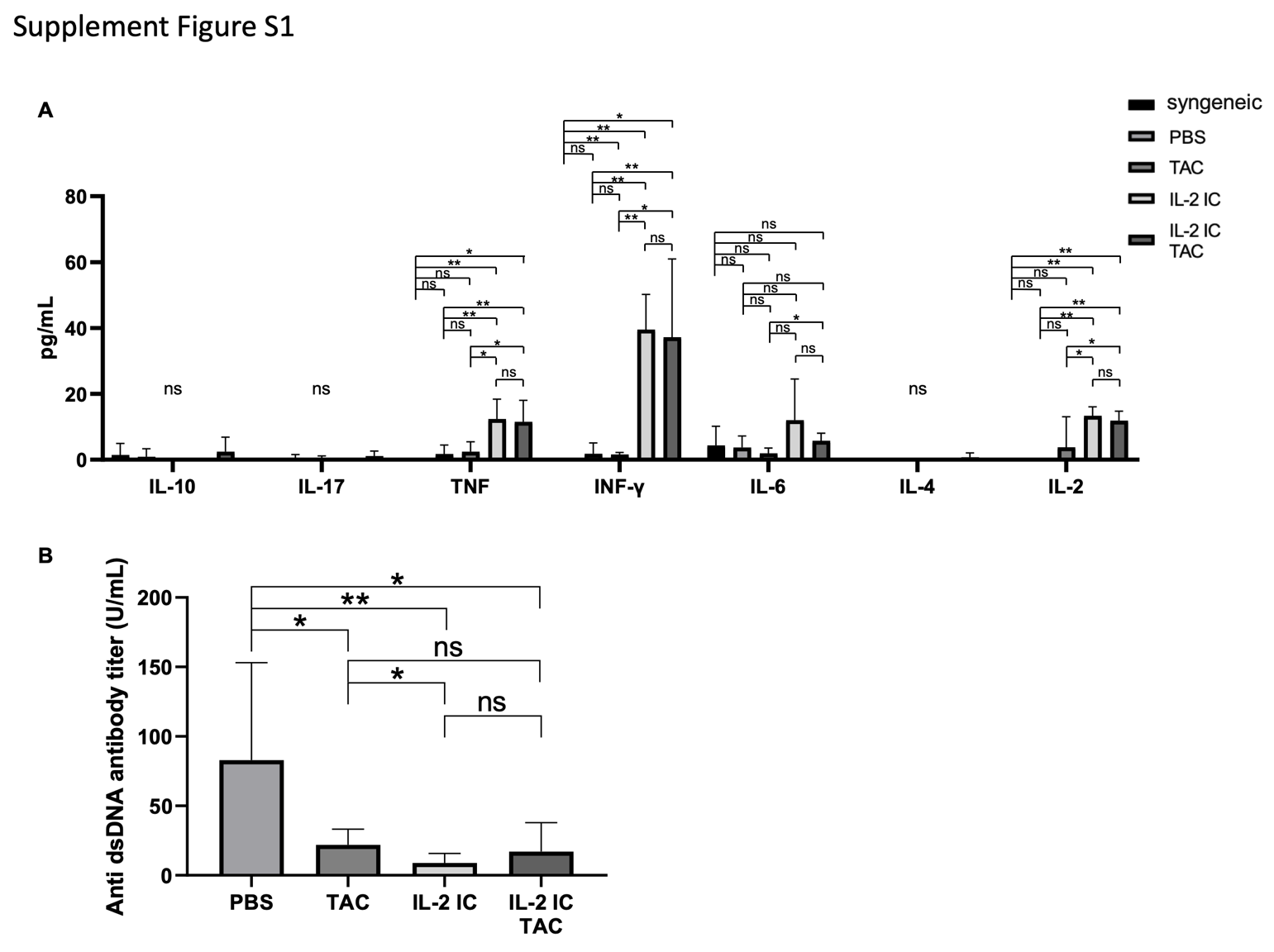
**Supplementary Figures**

**Supplementary Figure 1.** Concentration of serum cytokine and anti-dsDNA antibody titers in a prophylactic setting. (A) Serum cytokine levels were measured at 2 days after transplantation. Compiled data from two independent experiments are shown as the mean ± SD; n = 6 (syngeneic), 10 (PBS), 6 (tacrolimus), 6 (IL-2 IC), and 6 (IL-2 IC plus tacrolimus) mice/group. (B) Prophylactic treatment with tacrolimus, IL-2 IC, or IL-2 IC plus tacrolimus inhibits production of ant-dsDNA antibody. Serum anti-dsDNA antibody titer of host mice prophylactically treated with tacrolimus, IL-2, or IL-2 plus tacrolimus determined at two weeks after graft versus host disease induction. Compiled data from three independent experiments are presented as the mean ± SD; n = 9−13 mice/group. **p* < 0.05 and ***p* < 0.001, calculated using unpaired two-tailed Student’s *t*-test. ns: nonsignificant.


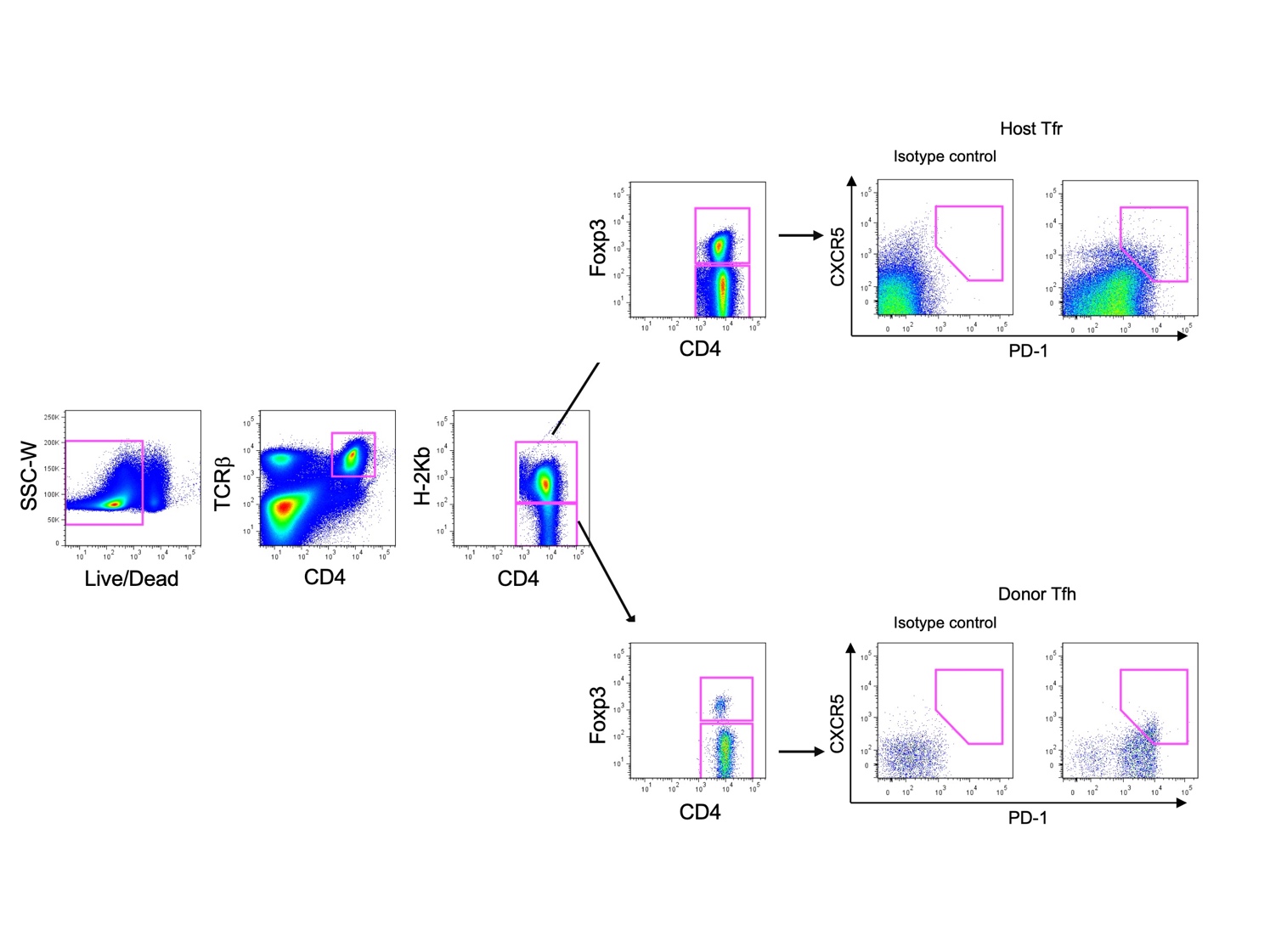


**Supplementary Figure 2.** Gating strategy for fluorescence-activated cell sorting analysis of follicular regulatory T cells (Tfrs) and follicular helper T cells (Tfhs).

**
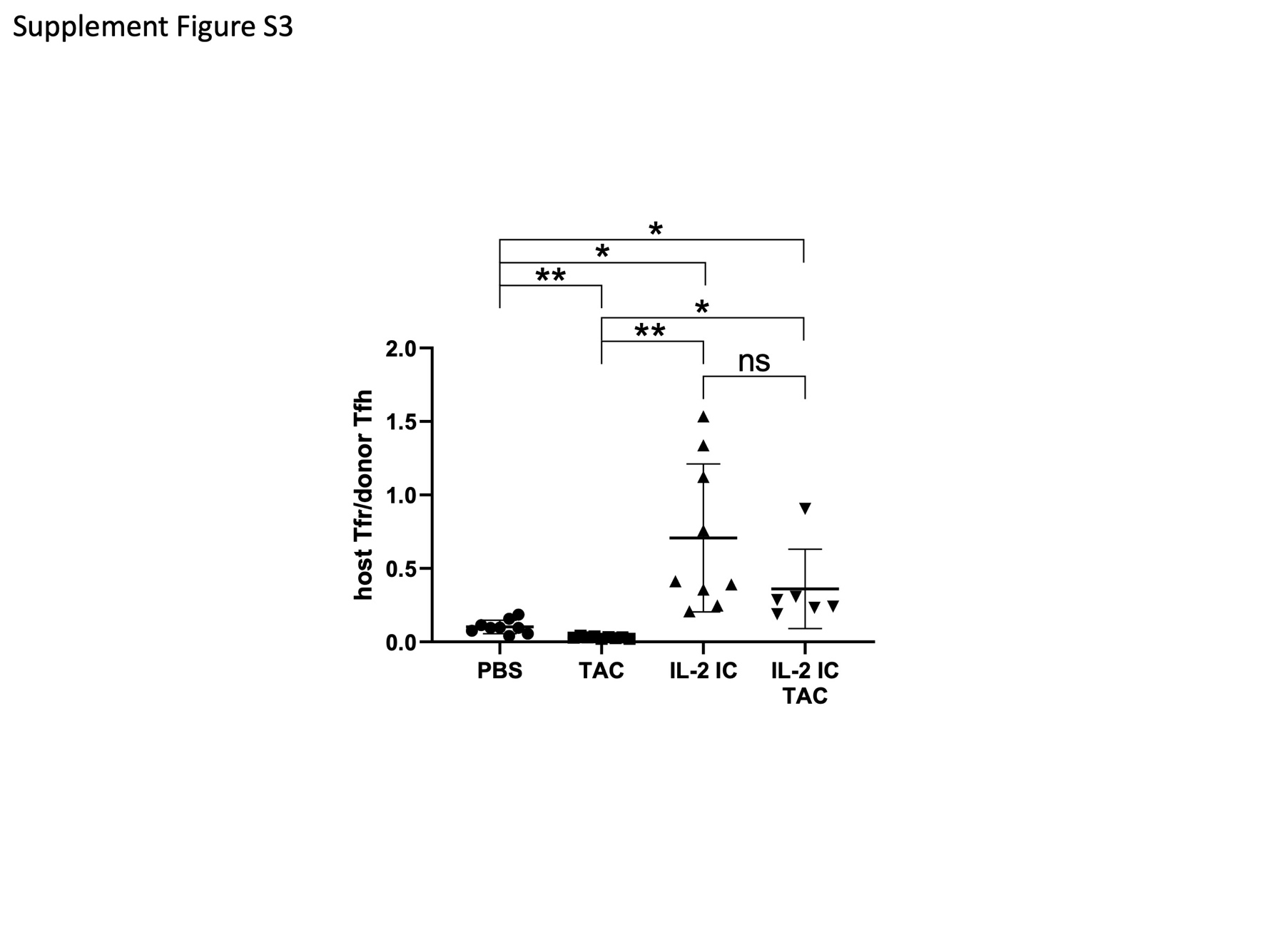
**

**Supplementary Figure 3.** Prophylactic treatment with IL-2 IC or IL-2 IC plus tacrolimus increased the Tfr/Tfh ratio in systemic erythematosus lupus (SLE)-like chronic graft versus host disease (GVHD) model host mice. Host-derived follicular regulatory T cells (Tfr)/donor-derived follicular helper T cell (Tfh) ratio in splenocytes harvested on day 30 after transplantation. Compiled data from five independent experiments are presented as the mean ± SD; n = 6−9 mice/group. **p* < 0.05 and ***p* < 0.001, calculated using unpaired two-tailed Student’s *t*-test. ns: nonsignificant.

**
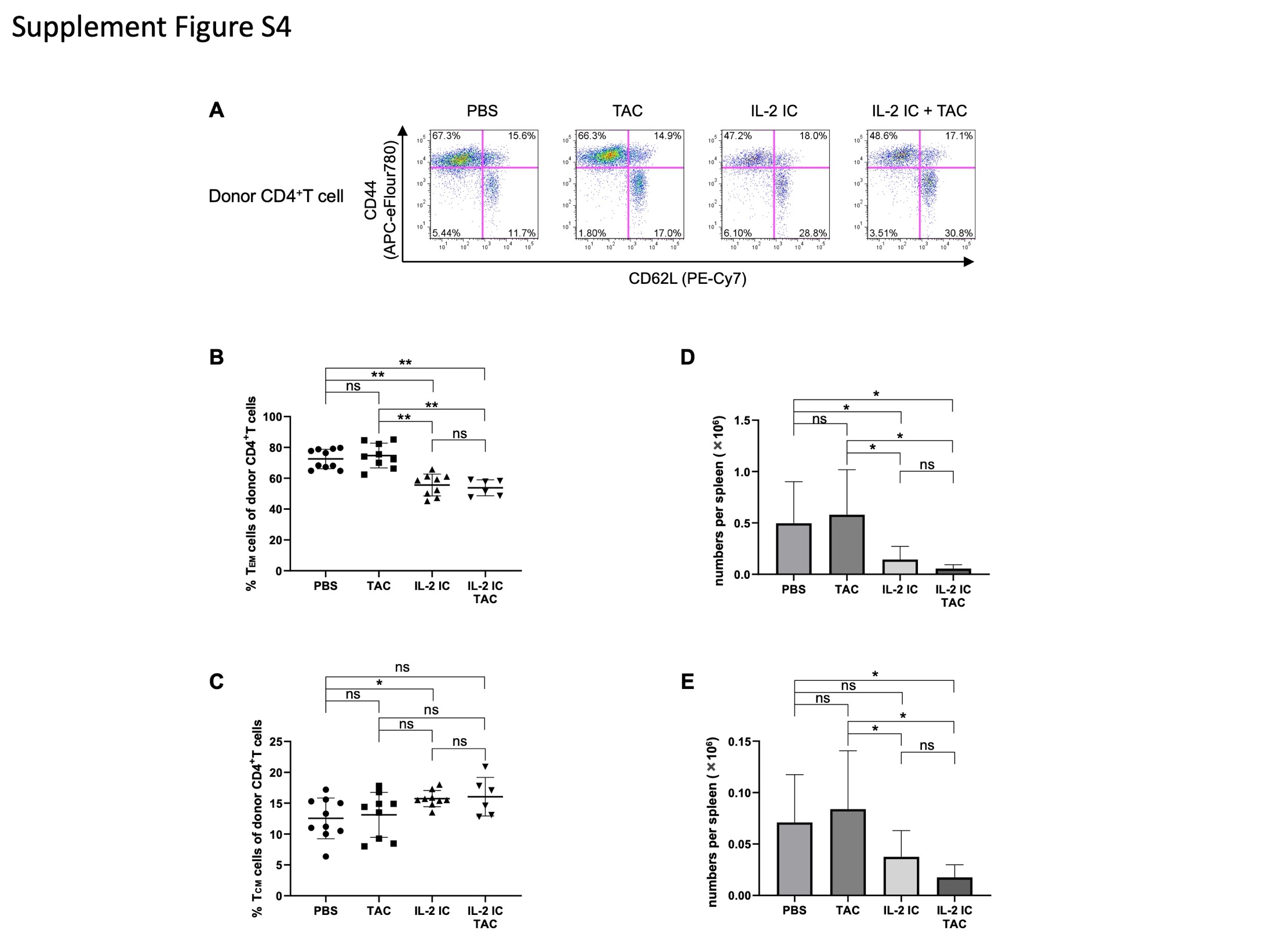
**

**Supplementary Figure 4.** Prophylactic treatment with IL-2 IC or IL-2 IC plus tacrolimus decreased donor-derived effector and memory CD4^+^ T cell levels in the systemic erythematosus lupus (SLE)-like chronic graft versus host disease (GVHD) model host mice. **(A)** Flow cytometry analysis of naïve and effector T cells in the splenic T cells at 7 days after transplantation. Representative fluorescence-activated cell sorting (FACS) plots of donor-derived cells gated on live CD4^+^TCRβ^+^ cells. **(B)** Proportion and **(C)** absolute number of effector memory T cells. **(D)** Proportion and **(E)** absolute number of central memory T cells. Compiled data from five independent experiments are presented as the mean ± SD; n = 6−10 mice/group. **p* < 0.05 and ***p* < 0.001, calculated using unpaired two-tailed Student’s *t*-test. ns: nonsignificant.

**
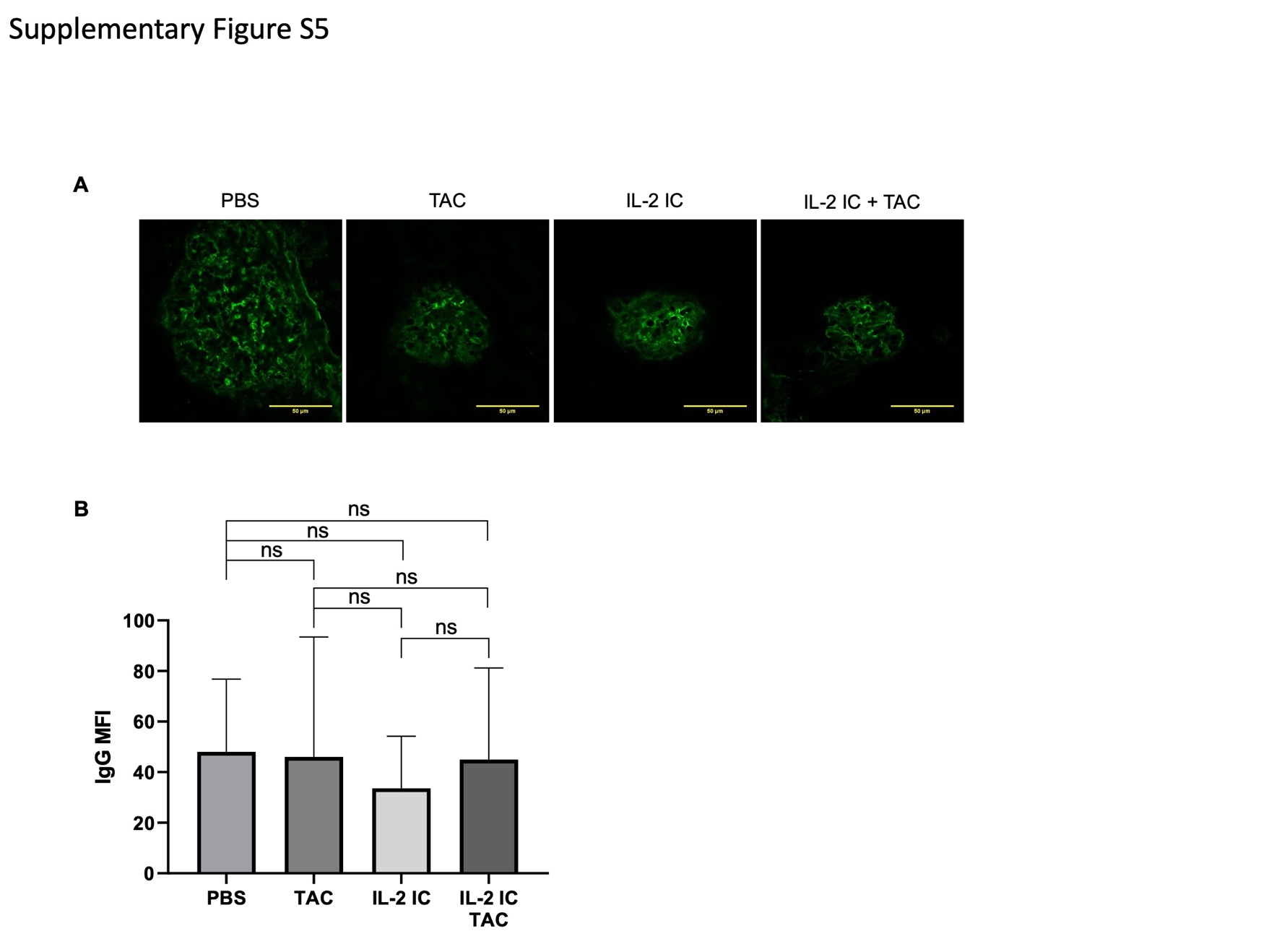
**

**Supplementary Figure 5.** Immuno-histochemical analysis of IgG deposition in the glomeruli of host mice. Kidneys were obtained from host mice at 30 days after transplantation. **(A)** Cryo-sections were prepared and stained with anti-IgG antibody. **(B)** Compiled mean fluorescence intensity data from two independent experiments are presented as the mean ± SD; n = 6 mice per group. ns: nonsignificant.

**
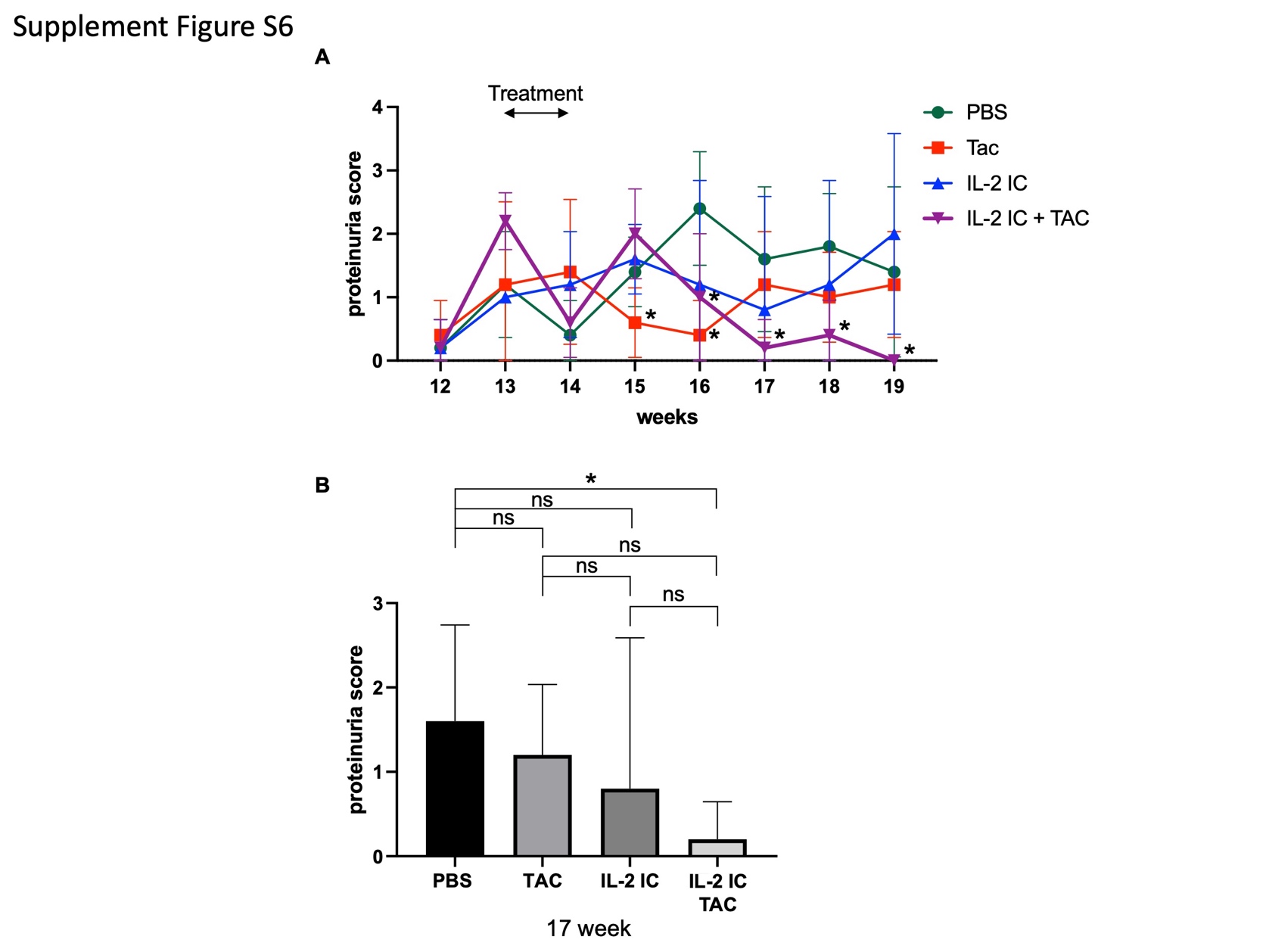
**

**Supplementary Figure 6.** Therapeutic treatment of MRL/lpr lupus mouse model. At 13 weeks of age, mice were treated with PBS, tacrolimus, IL-2 IC, or IL-2 IC plus tacrolimus. **(A)** Changes in urinary protein excretion levels by proteinuria score. A statistical comparison was conducted between mice treated with PBS and those treated with tacrolimus, IL-2 IC, or IL-2 IC plus tacrolimus at each time point. **(B)** Urinary protein excretion levels at 17 weeks of age. Data are presented as the mean ± SD; n = 5 mice/group. **p* < 0.05 calculated using unpaired, two-tailed Student’s *t*-test. ns: nonsignificant.

**
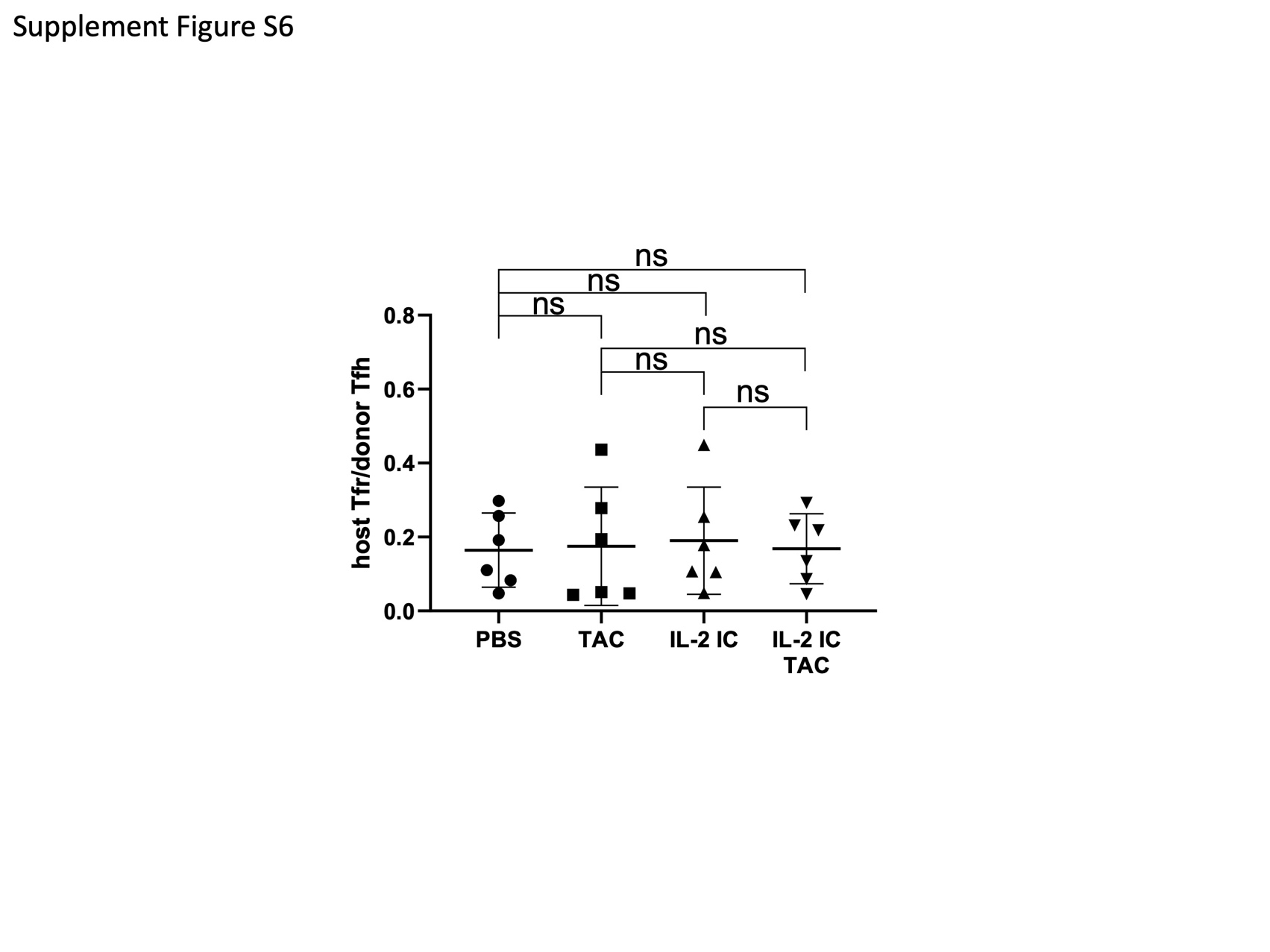
**

**Supplementary Figure 7.** Therapeutic treatment with tacrolimus, IL-2 IC, and IL-2 IC plus tacrolimus did not alter the Tfr/Tfh ratio in the systemic erythematosus lupus (SLE)-like chronic graft versus host disease (GVHD) model host mice. Host-derived follicular regulatory T cell (Tfr)/donor-derived follicular helper T cell (Tfh) ratio in splenocytes harvested on day 30 after transplantation. Compiled data from two independent experiments are presented as the mean ± SD; n = 6 mice/group. ns: nonsignificant.

**
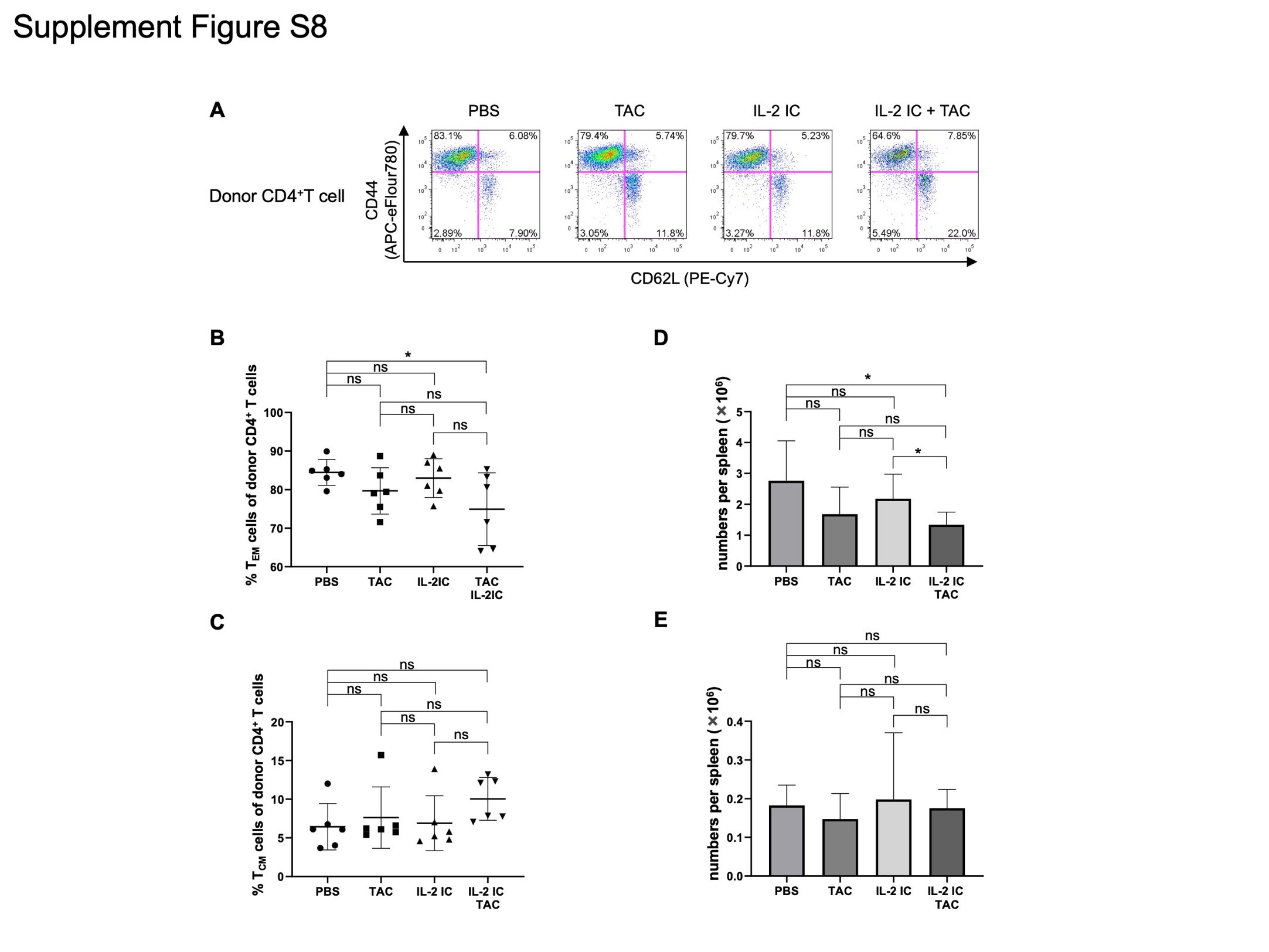
**

**Supplementary Figure 8.** Therapeutic treatment with IL-2 IC plus tacrolimus decreased donor-derived effector memory CD4^+^ T cell levels in systemic erythematosus lupus (SLE)-like chronic graft versus host disease (GVHD) host mice model. **(A)** Flow cytometry analysis of naïve and effector T cells in the splenic T cells at 30 days after transplantation. Representative fluorescence-activated cell sorting (FACS) plots of donor-derived cells. The plots were gated on live CD4^+^TCRβ^+^ cells. **(B)** Proportion and **(C)** absolute number of effector memory T cells. **(D)** Proportion and **(E)** absolute number of central memory T cells. Compiled data from two independent experiments are presented as the mean ± SD; n = 6 mice/group. **p* < 0.05 calculated using unpaired two-tailed Student’s *t*-test. ns: nonsignificant.

**
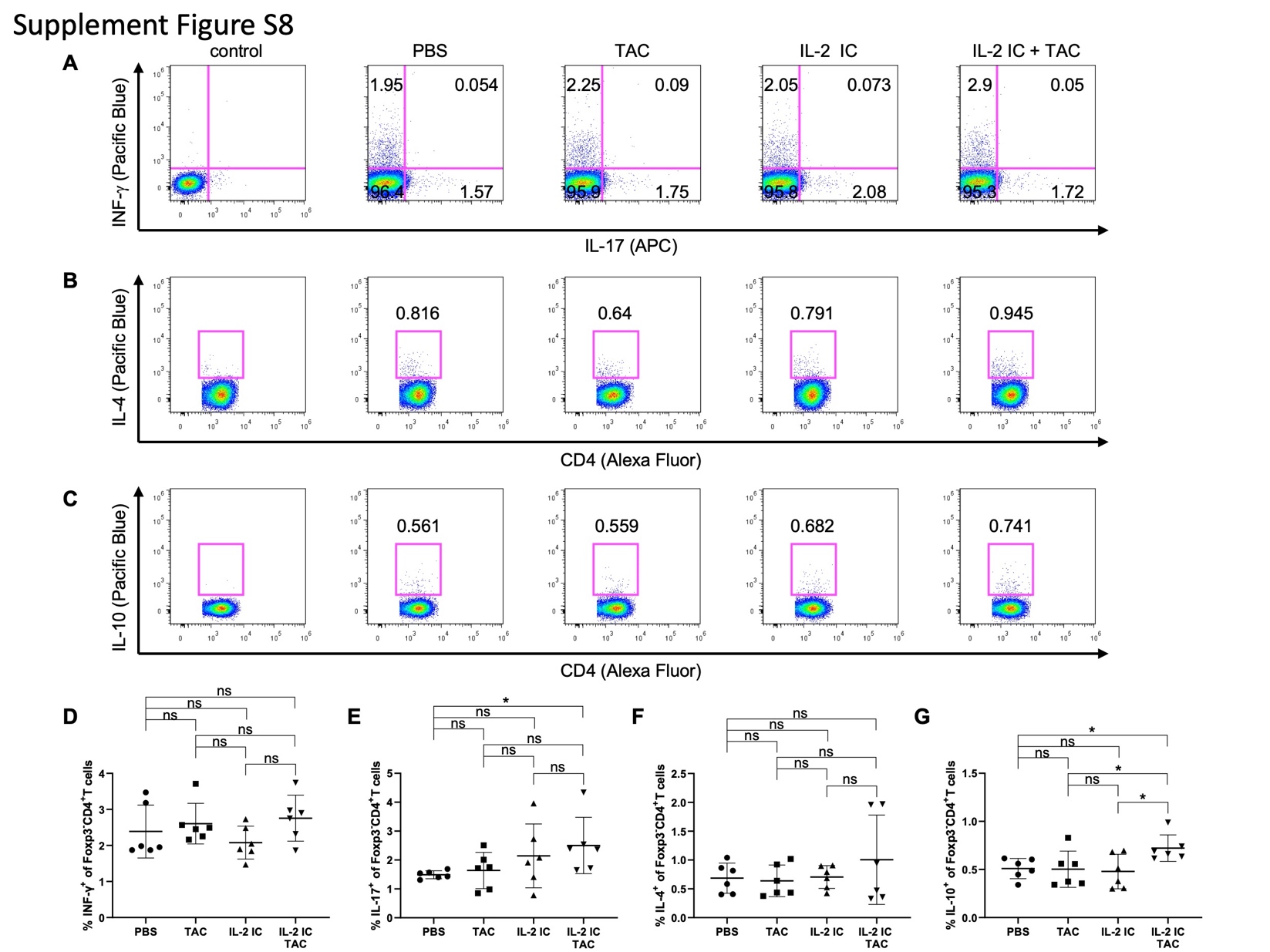
**

**Supplementary Figure 9.** Therapeutic treatment with IL-2 IC plus tacrolimus increased IL-17^+^ CD4^+^ and IL-10^+^ CD4^+^ T cells in the systemic erythematosus lupus (SLE)-like chronic graft versus host disease (GVHD) host mice model. At 5 weeks after GVHD induction, spleen cells were stimulated *in vitro*. The expression of IFN-γ, IL-17, IL-4, and IL-10 by Foxp3^-^CD4^+^ T cells was analyzed using flow cytometry. (A–C) The plots were gated on live CD4^+^ cells. Representative fluorescence-activated cell sorting (FACS) plots of two independent experiments and (D–G) compiled data of both experiments are represented as the means ± SD of n = 6 mice/group. **p* < 0.05 calculated using two-tailed Student’s *t*-test. ns: nonsignificant.
